# Markers of Vascular Function and Future Coronary Artery Disease Risk Among Malaysians with Individual Cardiovascular Risk Factors

**DOI:** 10.3390/biomedicines13040899

**Published:** 2025-04-08

**Authors:** Amilia Aminuddin, Nina Diyana Rusanuar, Md Rizman Md Lazin Md Lazim, Azizah Ugusman, Izzat Zulhilmi Abd Rahman, Kalaivani Chellappan, Mohd Shawal Faizal Mohamad, Wan Amir Nizam Wan Ahmad, Wan Yus Haniff Wan Isa

**Affiliations:** 1 Department of Physiology, Faculty of Medicine, Universiti Kebangsaan Malaysia, Kuala Lumpur 56000, Malaysia; ninadiyana@ukm.edu.my (N.D.R.); dr.azizah@hctm.ukm.edu.my (A.U.); izzat_zulhilmi@ukm.edu.my (I.Z.A.R.); 2 Department of Physiology, School of Medical Sciences, Health Campus, Universiti Sains Malaysia, Kota Bharu 16150, Malaysia; mdrizman@usm.my; 3 Department of Electrical, Electronic and Systems Engineering, Faculty of Engineering, Universiti Kebangsaan Malaysia, Bangi 43650, Malaysia; kckalai@ukm.edu.my; 4 Cardiology Unit, Department of Medicine, Hospital Canselor Tuanku Muhriz, Kuala Lumpur 56000, Malaysia; superwall81@gmail.com; 5 Biomedicine Programme, School of Health Sciences, Health Campus, Universiti Sains Malaysia, Kota Bharu 16150, Malaysia; wanamir@usm.my; 6 Department of Internal Medicine, School of Medical Sciences, Health Campus, Universiti Sains Malaysia, Kota Bharu 16150, Malaysia; wyhaniff@usm.my

**Keywords:** vascular function, coronary artery disease, pulse wave velocity, augmentation index, finger photoplethysmography, Framingham risk score

## Abstract

**Background/Objectives:** Vascular function measurements, including central parameters [pulse wave velocity (PWV) and augmentation index (AI)], as well as peripheral measures [finger photoplethysmography fitness index (PPGF)], have been introduced to detect early vascular damage associated with coronary artery disease (CAD) risk factors. This study aimed to compare peripheral and central vascular function marker levels among subjects with hypertension (HPT), dyslipidemia, and obesity. We also aimed to determine the relationship between these markers and CAD risk factors among these groups. **Methods:** A total of 320 subjects including healthy individuals and those with CAD risk factors were recruited. Peripheral vascular function was assessed using the PPGF, whereas central vascular markers included measurements of PWV and AI. The Framingham risk score (FRS) was calculated using an online calculator. **Results:** The mean age of the subjects was 33.73 ± 7.29 years. PWV and AI were significantly higher in HPT subjects (8.03 ± 1.40 m/s and 21.90% ± 10.57%) than the control. PPGF levels showed no significant differences between the groups. PWV was associated with FRS in the HPT and dyslipidemia groups, whereas AI was associated with FRS in the obese group. PPGF showed associations with PWV and AI in the dyslipidemia group. **Conclusions:** PWV and AI serve as robust macrovascular markers indicating arterial stiffness and systemic vascular resistance linked to CAD risk, while PPGF, as a microvascular marker, offers valuable insights into early endothelial dysfunction and microcirculatory anomalies, especially in dyslipidemia subjects.

## 1. Introduction

Cardiovascular disease (CVD) is the world’s leading cause of death, claiming 17.9 million lives annually and contributing to 31% of all global fatalities. Data show that more than 75% of CVD deaths occur in low- and middle-income countries, and 85% of all CVD deaths are caused by heart attacks and strokes. CVD encompasses a cluster of diseases involving the heart and blood vessels, such as atherosclerotic cardiovascular disease, cerebrovascular disease, valvular disease, and several other conditions [1]. The rise in CVD risk factors, such as diabetes mellitus (DM), hypertension (HTN), dyslipidemia, and obesity, is contributing to the increasing incidence of CVD [2]. These risk factors contribute to vascular damage and increase the risk of heart attack and stroke [3].

In Malaysia, the prevalence of these risk factors is high and continues to rise, leading to an increased rate of CAD. Additionally, Malaysians tend to develop CAD at a younger age than Western populations [4]. Lifestyle factors such as high carbohydrate intake, excessive consumption of saturated fats, and a sedentary lifestyle may further exacerbate vascular dysfunction [5,6]. Given these population-specific characteristics, evaluating vascular function markers in the Malaysian context is crucial to the early detection and prevention of CAD.

Vascular function can be assessed through invasive and non-invasive methods. Invasive assessments such as intravascular ultrasound and coronary angiography provide direct measurements of arterial health but are often impractical for large-scale studies owing to their cost, complexity, and associated risks. Instead, non-invasive techniques offer safer and more feasible options for early vascular assessment and population-based research.

Vascular assessments can also be categorized based on their focus on central or peripheral circulation. Central vascular function primarily reflects large arterial stiffness and systemic hemodynamics. It can be evaluated using pulse wave velocity (PWV), augmentation index (AI), cardio-ankle vascular index, cardiac magnetic resonance, and magnetic resonance imaging of aortic diameter [7]. Peripheral vascular function reflects microvascular health and endothelial function. It can be assessed using methods such as ankle-brachial index, doppler ultrasound pulse volume recording, flow-mediated dilation, reactive hyperemia index, pulse wave analysis, and finger photoplethysmography fitness index (PPGF) [8,9,10]. PWV and AI are well-established indicators of cardiovascular risk and are commonly used in epidemiological studies [11]. PPGF is reportedly correlated with arterial stiffness and endothelial function, both of which are relevant to cardiovascular risk assessment [12]. Evaluating central and peripheral vascular function provides a more holistic understanding of vascular health because abnormalities in either system may contribute to cardiovascular pathology. Although central arterial stiffness is linked to increased afterload on the heart and HPT, peripheral vascular dysfunction may reflect early endothelial changes associated with metabolic and inflammatory disorders [7,13].

PWV is calculated as the distance traveled by the pulse wave divided by the time taken to travel that distance (path length) [14,15]. AI is another parameter for vascular function that is based on the reflection of pressure waves, and it is accepted as a measure of systemic arterial stiffness [16,17]. PPGF is a technique that uses optical methods to measure blood volume changes in the microvascular space (arterioles) of the finger [18]. It is calculated by comparing one subject’s pulse to a reference pulse [19]. The reference is a healthy 19-year-old subject free of CVD risk factors such as HPT, dyslipidemia, smoking, and other clinically diagnosed chronic diseases [20]. The sensitivity of PPGF in identifying subjects with at least one risk factor exceeds 80% [14].

Many studies have examined vascular function markers among individuals with CAD risk factors and healthy subjects, but no studies have compared these markers across different individual risk factors. Thus, the present study aimed to compare the levels of vascular function markers among subjects with HPT, dyslipidemia, and obesity and to determine the relationship between vascular function markers and CAD risk factors in these groups.

## 2. Materials and Methods

Subjects were recruited through a convenience sampling technique and were gathered from health-screening programs held in Klang Valley, Malaysia. This study was approved by the Ethics Research Committee, Faculty of Medicine, Universiti Kebangsaan Malaysia (UKM PPI/111/8/JEP-2018-328). The inclusion criteria were individuals aged 18–64 years who were healthy or had cardiovascular risk factors such as HPT, dyslipidemia, or obesity. Each participant was assigned to only one group based on predefined criteria, ensuring that no individual qualified for multiple groups. The classification process was carefully structured to prevent overlap and maintain clear distinctions between groups.

Those with any chronic inflammatory diseases, joint, or muscle illness (e.g., rheumatoid arthritis, systemic lupus erythematosus, inflammatory bowel disease) were excluded. All subjects underwent a detailed clinical history, physical examination, and electrocardiography [ECG (KENZ^®^ Cardico 1215, Nagoya, Japan)]. The clinical history included an evaluation of key cardiovascular symptoms such as chest pain, shortness of breath, palpitations, dizziness, and syncope. Additionally, past medical history was reviewed, including any previous diagnosis of myocardial infarction (MI), peripheral artery disease (PAD), hypertension, diabetes mellitus, dyslipidemia, or other cardiovascular risk factors. A history of smoking, family history of premature CAD, and medication use were also documented (if any). Only individuals with normal physical examination findings and ECG results were included in the study. Participants with ECG abnormalities suggestive of CAD were excluded to ensure that the study population comprised individuals without evident cardiac ischemia. No additional diagnostic imaging for CAD was performed. Specific diagnostic tests for PAD, such as Doppler ultrasound or ankle-brachial index measurement, were not performed. We also did not assess the peripheral pulses for PAD. Consequently, PAD was not systematically assessed in this study.

Fasting blood glucose (FBS) was measured to assess diabetes mellitus (DM) status [>7.0 mmol/L (126 mg/dL)]. While FBS is a widely used diagnostic marker, we acknowledged that glucose levels can fluctuate depending on fasting duration. HbA1c, which provides a better reflection of long-term glycemic control, was not measured in this study. Subjects with DM were excluded since they may have advanced vascular damage compared with those with other CAD risk factors. HPT was defined as systolic blood pressure (SBP) ≥ 140 mmHg or diastolic blood pressure (DBP) ≥ 90 mmHg. Dyslipidemia was defined as total cholesterol (TC) > 5.2 mmol/L (200 mg/dL) in accordance with clinical guidelines. Obesity was defined as BMI ≥ 27.5 kg/m^2^ in accordance with the WHO and regional guidelines for Asian populations, where a lower BMI threshold is associated with increased cardiovascular risk [21]. Subjects needed to fast for 8 h prior to the measurement.

### 2.1. Weight and Height Measurement

Weight was measured using a digital scale (SECA, Hamburg, Germany). Height was measured using a stadiometer attached to the wall (SECA, Hamburg, Germany). BMI was calculated as weight/height^2^ (kg/m^2^).

### 2.2. Blood Pressure (BP) Measurement

The baseline brachial SBP (bSBP) and brachial DBP (bDBP) of subjects were measured once using a digital BP monitor (Omron HEM 8712, Kyoto, Japan) while they were in a sitting position after resting for 5 min. For individuals with initially elevated BP readings, two to three additional measurements were taken after a few minutes of rest. The final BP classification was based on the average of these repeated measurements to ensure accuracy and minimize misclassification. However, repeated BP assessment on multiple occasions would provide a more precise classification.

### 2.3. Blood Parameters Measurement

Venous blood (10 mL) was withdrawn from the antecubital vein. Blood was collected in plain, EDTA, and fluoride-containing tubes. All blood specimens were sent to Gribbles Pathology laboratory (Petaling Jaya, Selangor, Malaysia) for analysis of biochemical parameters. They included full blood count, which was equivalent to the complete blood count, consisting of white blood cell count, red blood cell count, hemoglobin, and platelet count, fasting serum lipid, equivalent to the lipid profile, included TC, high-density lipoprotein (HDL), triglycerides (TG), and low-density lipoprotein (LDL, calculated). FBS was equivalent to fasting blood glucose. Renal function test included blood urea nitrogen and serum creatinine. Liver function test included aspartate aminotransferase, alanine aminotransferase, alkaline phosphatase, total bilirubin, and direct bilirubin. All listed tests were measured using enzymatic methods (Advia 2400 Chemistry Analyzer, Siemens, Tokyo, Japan). The hs-CRP level was determined using the immunology method (Advia, 2400 Chemistry Analyzer, Siemens, Tokyo, Japan).

### 2.4. Physical-Activity Assessment

The International Physical Activity Questionnaire (IPAQ) was used to assess physical activity. The IPAQ is available in Malay and English languages and has been validated [22]. Short-term evaluation IPAQ consists of an assessment of activities like mild activities (walking), moderate activities (mild cycling and mopping floor), and heavy activities (jogging and swimming). Mild activity is equivalent to 3.3 metabolic equivalents (METS), moderate activities to 4 METS, and heavy activities to 8 METS. One METS is equivalent to the energy consumption at rest, equal to 3.5 mL O_2_/kg/min. The duration of activities (in minutes) and frequency of activities per week were recorded. Physical activity was calculated as the total METS [(mild/moderate/heavy) × duration per day (minute) × frequency per week].

### 2.5. Framingham Risk Score (FRS) Calculation

FRS was measured using an online calculator from https://www.soapnote.org/cardiovascular/framingham/ (accessed on 1 January 2023). The calculation of FRS was based on the information on the presence of CVD in a subject including clinical CAD, symptomatic carotid artery disease, peripheral arterial disease, abdominal aortic aneurysm, and DM. Other required parameters include gender, age, TC level, HDL level, smoking status, HPT status (treated or untreated), and SBP level. BMI was not included in the FRS calculation.

### 2.6. Measurement of PPGF

PPGF was recorded using a finger PPG sensor with subjects positioned supine in a room maintained at 20–25 °C. Subjects fasted for at least 8 h before the measurements. They rested for 5 min. Following the rest period, a finger probe was applied to the right index finger of each subject, and measurements were taken for at least 120 s.

### 2.7. Measurement of Central BP (CBP), PWV, and AI

CBP (cSBP and cDBP), PWV, and AI were measured using an arteriograph device (TensioMed Ltd., Budapest, Hungary). The arteriograph was equipped with an inflatable cuff attached to the subject’s upper arm and inflated to a pressure of 35 mmHg above the subject’s SBP. Pressure variations were detected with a pressure sensor, and the signal was transferred into a computer. PWV was calculated based on the production of two systolic peaks: the first peak (P1) resulted from the ejection of systolic volume in the aorta, whereas a slightly lower second peak (P2) was produced by the reflection of pressure waves from peripheral arteries. PWV was calculated as [the distance from the jugulum to the symphysis (m)/return time (s)/2] [23]. AI was calculated as [(second systolic peak–first systolic peak)/pulse pressure × 100].

### 2.8. Statistical Analysis

Data analysis was conducted using the Statistical Package for the Social Sciences (IBM SPSS Statistics version 26). The data for all subjects were categorized into groups with or without individual CVD risk factors; normal, HPT, dyslipidemia, and obesity. ANOVA and post hoc Tukey tests were used to compare the value of vascular function markers between groups. The comparison of PWV included adjustments for age, whereas the comparison of AI included adjustments for age, heart rate (HR), and height. These adjustments were made by ANCOVA. The relationship between the central and peripheral vascular function markers among the subjects with HPT, subjects with dyslipidemia, and obese subjects was assessed using correlation tests. Pearson correlation was used for all parameters except for CRP and PA in subjects with dyslipidemia and obesity, for which Spearman correlation was used.

## 3. Results

### 3.1. Subject Characteristics

Initially, 520 subjects were recruited; however, after exclusions, the final sample consisted of 320 subjects. The characteristics of the subjects are summarized in Table 1. They were normal group (N = 125), HPT group (N = 34), dyslipidemia group (N = 109), and obese group (N = 52). The mean age of all subjects was 33.73 ± 7.29. None of the subjects were on any medication. Obese subjects had higher BMI than normal subjects, HPT subjects, and subjects with dyslipidemia (*p* < 0.001). HPT subjects had a higher mean value of bSBP than normal subjects, whereas subjects with dyslipidemia and obesity had bSBP in the pre-HPT range. The median CRP was highest in subjects with obesity and was within moderate risk in HPT subjects and subjects with dyslipidemia. Obese subjects had higher CRP than normal (*p* < 0.001), dyslipidemia (*p* < 0.001), and HPT (*p* = 0.007). The FRS average score indicated low risk for all groups. Subjects with dyslipidemia had a higher FRS than normal (*p* < 0.001), HPT (*p* = 0.02), and obese (*p* < 0.001) subjects.

### 3.2. Central and Peripheral Vascular Function Markers

Table 2 shows the comparison of central and peripheral vascular function markers between subjects with HPT, dyslipidemia, obesity, and healthy subjects using ANOVA and post hoc analysis. No difference in PPGF existed between the groups. Subjects with HPT had higher PWV than healthy subjects (*p* < 0.001), subjects with dyslipidemia (*p* = 0.001), and obese subjects (*p* = 0.038). After adjusting for age, subjects with HPT had higher PWV than healthy subjects (*p* = 0.002) and subjects with dyslipidemia (*p* = 0.003), with no significant difference compared with obese subjects. Subjects with HPT (*p* < 0.001) and subjects with dyslipidemia (*p* = 0.005) had higher AI than healthy subjects. Subjects with HPT also had higher AI than obese subjects (*p* = 0.046). After adjusting for age, HR, and height, subjects with HPT had higher AI than healthy subjects (*p* = 0.003). However, after adjusting for age, HR, and height, the AI value was not significantly different between dyslipidemia and normal groups and HPT and obese groups.

### 3.3. Correlation Between Vascular Function Markers and CVD Risk Factors

Table 3 shows the correlation results between vascular function markers and CVD risk factors among subjects with HPT, dyslipidemia, and obesity. Variations in each vascular marker existed across the groups. PWV had a consistent significant association with blood pressure in all the groups. PWV was also associated with FRS in the HPT and dyslipidemia groups. In the HPT group, PWV was associated with numerous CVD risk factors compared with the other groups. AI had a persistent association with BP across the groups and was associated with FRS in the obese group. PPG was associated with BP, PWV, and AI in the dyslipidemia group.

## 4. Discussion

We compared the levels of central and peripheral vascular markers between normal group and groups with HPT, dyslipidemia, and obesity. AI and PWV are well established to be elevated in individuals with HPT, and FRS scores tend to be higher in those with dyslipidemia. However, our study aimed to evaluate these markers within a Malaysian cohort, where population-specific differences may exist. Additionally, by comparing AI, PWV, and PPGF across different cardiovascular risk groups, we provided insights into their relative utility for early vascular dysfunction screening.

### 4.1. PWV in Various CVD Risk Factors

After adjusting for age, the mean PWV was recorded to be higher in the HPT group, compared to the mean values of the normal and dyslipidemia groups. Increased PWV among subjects with HPT compared with normal subjects has also been found in previous studies [24,25]. This increase may be due to several mechanisms. In HPT, injury to the arterial wall can cause chronic inflammation [26,27], which leads to the remodeling of the vascular wall [27,28]. This situation involves the occurrence of fragmentation and loss of elastin fibers, as well as the accumulation of collagen fibers that become progressively stiffer in the arterial wall [29,30,31]. The production of stiffer blood vessels causes the PWV to increase.

Higher PWV in hypertensive individuals suggests early arterial stiffening, which increases the risk of MI, heart failure, and stroke [32]. Given its correlation with the FRS, PWV can serve as an additional tool for cardiovascular risk stratification, particularly in younger individuals where traditional risk scores may underestimate risk [33]. Incorporating PWV into routine assessments may guide treatment strategies because antihypertensives such as ACE inhibitors and ARBs have been shown to improve arterial compliance [34].

Additionally, lifestyle modifications such as sodium reduction, weight management, and structured exercise may help reduce arterial stiffness and long-term cardiovascular risk. In Malaysia, where dietary sodium intake is often high, physicians should emphasize nutritional counseling, encouraging patients to adopt a balanced diet low in processed foods and rich in fruits, vegetables, and whole grains [35]. Given that a 1 m/s increase in PWV has been linked to a 15% higher risk of cardiovascular events, routine PWV monitoring in hypertensive patients may aid in early intervention [36]. Physicians should also consider integrating culturally tailored interventions, such as community-based exercise programs and weight management strategies, to improve adherence and long-term cardiovascular outcomes.

For obese subjects, the results showed no significant difference in PWV between obese and normal subjects. This finding was in line with that of Desamericq et al. [37]. However, Lurbe et al. [38] found that PWV is significantly lower in obese subjects than in normal ones. Conversely, studies by Kulsum-Mecci et al. [39] and Son et al. [40] found that PWV is significantly higher in obese subjects than in normal ones. The differences in these results may be attributed to variations in age, BMI, ethnicity, dietary patterns, physical activity, life stress, type of jobs, and the presence of other risk factors. For example, a meta-analysis study has shown that the association between arterial stiffness and body fat showed a negative relationship until middle age, after which it became a positive relationship in older age [41].

Obesity is also associated with lower peripheral vascular resistance, which may not affect PWV [42,43]. However, this relationship is more complex and cannot be generalized, particularly in individuals with normotensive versus hypertensive obesity. The lack of a significant PWV increase in our obese cohort may be due to compensatory vascular adaptations, such as lower peripheral vascular resistance and maintained arterial elasticity in younger obese individuals. Obesity alone may not be sufficient to induce arterial stiffness unless accompanied by metabolic dysfunction, hypertension, or prolonged disease duration [44]. Other contributing factors, such as autonomic regulation, endothelial dysfunction, and inflammation, may also play a role [44]. This underscores the importance of considering obesity-related factors such as insulin resistance, inflammation, and lipid abnormalities when evaluating vascular risk. Future studies should investigate whether PWV changes over time in obese individuals, particularly those with metabolic syndrome or hypertension, to better understand its role in early cardiovascular risk assessment.

After age adjustment, no significant difference in PWV was found between subjects with dyslipidemia and normal subjects. However, previous studies have found that PWV is significantly higher in subjects with dyslipidemia than in normal subjects [45,46,47,48,49]. Our finding was inconsistent with previous ones possibly due to several factors: (i) our study mostly involved subjects who were newly diagnosed, so damage to the vessels may have occurred at an early stage; (ii) the presence of other risk factors besides dyslipidemia in previous studies [46]; or (iii) comparison of PWV was not standardized for age as a confounder [47,49].

### 4.2. AI for Various CVD Risk Factors

Our study found that after adjusting for age, HR, and height, AI was significantly higher in HPT subjects than in normal ones. Previous studies also found significantly higher AI in HPT subjects than in normal ones [25,50]. The increase in AI values among HPT subjects may be due to an increase in PWV, which can affect the value of AI [51,52]. Additionally, HPT is associated with increased total peripheral resistance, which may increase AI [53].

For dyslipidemia and obese subjects, our study found that after adjusting for confounders, their AI did not differ from those of normal subjects. Previous studies have also found no difference in AI between dyslipidemia and normal subjects [47,54] and between obese and normal subjects [38,40]. Conversely, a previous study has shown that AI increases in subjects with dyslipidemia [49] and decreases in obese subjects [55] compared with normal ones.

The current study extended our previous research on AI among relatively young subjects. Our previous study among Malaysian young men showed that AI increases among those with at least two risk factors compared to healthy subjects [56]. No difference in AI exists between the one-risk-factor group and healthy subjects. In that study, the risk factors were mixed and not focused on any specific one. In contrast, the current study focused on specific risk factors and found that AI was elevated in the HPT group, indicating that AI may be a sensitive marker for HPT.

The higher AI observed in hypertensive individuals suggested increased vascular stiffness and peripheral resistance, leading to elevated central aortic pressure. This increased afterload on the heart can accelerate left ventricular hypertrophy, reduce coronary perfusion, and contribute to diastolic dysfunction and heart failure [57]. Given its role in hemodynamic stress, AI may provide additional insights into cardiovascular burden beyond conventional BP measurements. Monitoring AI in hypertensive patients can help identify those at greater risk of adverse outcomes, allowing for more tailored management strategies. Pharmacological interventions that improve arterial elasticity such as angiotensin converting enzymes inhibitors, angiotensin receptor blockers, and vasodilators, along with structured exercise programs, may help reduce AI and mitigate cardiovascular complications.

### 4.3. PPGF for Various CVD Risk Factors

The assessment of PPGF in this study showed no significant differences across subjects with HPT, dyslipidemia, obesity, and healthy controls. PPGF, derived from PPG signals, reflects blood volume changes in microvascular beds and provides insights into microvascular function and endothelial health. Unlike PWV and AI, which measure pressure-wave transmission and reflection associated with macrovascular stiffness, PPGF focuses on microcirculatory dynamics.

Meanwhile, previous studies have suggested the utility of PPGF in detecting vascular abnormalities [58]; the lack of significant findings in this study may be due to its sensitivity to early-stage disease where microvascular damage remains minimal or undetectable. The exclusion of diabetic subjects [59], who are known to present with more advanced microvascular dysfunction, may have further limited the sensitivity of PPGF in this cohort. Additionally, external factors such as skin properties, hydration status, and temperature, which influence PPGF measurements, were not fully controlled in this study.

### 4.4. Associations Between Vascular Markers and Other CVD Risk Factors

The results of the association study revealed that PWV was correlated with BP parameters in all groups. This finding supported a study by Cecelja and Chowienczyk [60] who found that PWV was more closely associated with BP parameters than other risk factors such as lipid level or glucose. Additionally, PWV was correlated with numerous CVD risk factors and the FRS, especially in HPT and dyslipidemia groups. This finding suggested the value of PWV as a sensitive marker of vascular damage in both groups.

Our study also found that the PPGF was associated with the AI and PWV in subjects with dyslipidemia. Previous studies have also found that PPGF is associated with PWV among subjects with CVD risk factors [12,61]. Thus, PPGF can serve as a valuable complementary marker for detecting lipid-related microvascular dysfunction. This potential should be further explored in studies involving broader populations, including those with more advanced disease stages. Integrating PPGF with established macrovascular markers may enhance CAD risk detection, particularly for individuals with early endothelial dysfunction related to dyslipidemia. As for AI, this marker also had a consistent association with BP across all groups and had significant associations with various CVD risk factors in the HPT and dyslipidemia groups. This finding suggested the usefulness of this marker in these groups. The current finding extends our previous study that emphasizes the use of AI as a sensitive marker of vascular damage among the young population with CVD risk factors [56].

### 4.5. Limitations

This study had several limitations. The age of subjects in each group was not standardized owing to the difficulty in recruiting subjects. Moreover, data on the duration of diseases among subjects were incomplete and were not accounted for as confounders. An equal or similar age and number of subjects in each risk factor group may generate more accurate results that represent the targeted study population. This study further used a convenience sampling technique, which may introduce selection bias. However, strict inclusion criteria and standardized assessments were used to reduce potential bias. Future studies with larger, randomized cohorts are recommended to confirm our findings. CAD or PAD was also not confirmed using angiography, which may limit the accuracy of CVD status. Future studies incorporating imaging-based validation such as coronary angiography can provide additional confirmation of these findings. Another limitation is that the FRS calculation in this study did not incorporate body mass index (BMI) as a risk factor. BMI is known to influence cardiovascular risk through mechanisms such as HPT, dyslipidemia, and insulin resistance, but it is not included in the standard FRS calculation. Given the rising prevalence of overweight and obesity in the Malaysian population, this omission may impact risk estimations, potentially underestimating cardiovascular risk in individuals where obesity plays a significant role. Future research should explore whether integrating BMI into existing risk prediction models can improve accuracy, particularly in populations with high obesity rates. Lastly, hypertriglyceridemia was not considered as an exclusion criterion, despite its established role as a cardiovascular risk factor, particularly through elevated very-low-density lipoproteins (VLDL) [62]. As a result, variations in triglyceride levels among participants may have influenced the outcomes, and future studies should consider stratifying or adjusting for hypertriglyceridemia to better assess its independent effects on cardiovascular risk.

## 5. Conclusions

The hypertensive group had higher PWV and AI than the normotensive group. From a clinical perspective, integrating PWV and AI measurements into routine assessments can facilitate the earlier detection of vascular dysfunction, allowing for targeted interventions such as antihypertensive therapy selection, structured exercise programs, and metabolic risk management. Additionally, PWV and AI may be particularly useful in identifying vascular dysfunction in individuals without a formal diagnosis of hypertension. Despite being classified as normotensive, some individuals may exhibit elevated PWV and AI, suggesting early vascular changes that could predispose them to future hypertension and cardiovascular events. Therefore, closer monitoring of normotensive individuals with elevated PWV and AI may help identify those at risk of developing hypertension and enable early preventive measures. In addition, the correlation between PPGF and PWV/AI in dyslipidemic subjects suggested a possible role as vascular markers and thus warrants further exploration. Future research should explore the long-term prognostic value of these markers and their integration into existing cardiovascular risk models to enhance early intervention efforts.

## Figures and Tables

**Table 1:** Characteristics of subjects in each group.

	Normal (N = 125)	HPT (N = 34)	Dyslipidemia (N = 109)	Obese (N = 52)
Age (year) ^a^	30.39 ± 5.50	35.62 ± 8.24 ^b^***	34.09 ± 7.82 ^e^***	33.00 ± 6.08
Height (cm) ^a^	159.80 ± 7.97	159.44 ± 6.04	159.18 ± 7.39	158.63 ± 5.94
Weight (kg) ^a^	56.85 ± 10.06	61.71 ± 8.33 ^d^***	58.41 ± 10.08 ^g^***	79.62 ± 11.50 ^f^***
BMI (kg/m^2^) ^a^	22.16 ± 3.01	23.65 ± 2.06 ^d^***	22.88 ± 2.71 ^g^***	32.77 ± 4.77 ^f^***
bSBP (mmHg) ^a^	119.70 ± 9.62 ^b^***	151.74 ± 12.93 ^d^***	121.99 ± 9.86 ^c^***	124.62 ± 8.38 ^f^*
bDBP (mmHg) ^a^	68.75 ± 8.16	86.97 ± 12.80 ^b^***	70.71 ± 8.30 ^c^***	71.40 ± 7.74 ^d^***
HR (bpm) ^a^	69.49 ± 10.73	70.53 ± 8.27	69.53 ± 10.49	71.33 ± 8.78
FBS (mmol/L) (mg/dL) ^a^	4.53 ± 0.34 (81.61 ± 6.12)	4.66 ± 0.50 83.95 ± 9.00	4.63 ± 0.40 (83.41 ± 7.21)	4.70 ± 0.43 ^f^* (84.67 ± 7.75) ^f^*
TC (mmol/L) (mg/dL) ^a^	4.47 ± 0.53 (172.83 ± 20.49)	4.53 ± 0.36 ^c^*** (175.15 ± 13.92) ^c^***	5.92 ± 0.84 ^e^*** (228.9 ± 32.48) ^e^***	4.44 ± 0.57 ^g^*** (171.67 ± 22.04) ^g^***
TG (mmol/L) (mg/dL) ^a^	0.76 ± 0.32 (67.31 ± 28.34)	1.09 ± 0.69 ^b^** (96.54 ± 61.11) ^b^**	1.08 ± 0.54 ^e^*** (96.66 ± 47.83) ^e^***	1.11 ± 0.67 ^f^*** (98.31 ± 59.34) ^f^***
HDL (mmol/L) (mg/dL) ^a^	1.46 ± 0.32 ^e^** (56.45 ± 12.37) ^e^**	1.42 ± 0.32 ^c^** (54.91 ± 12.37) ^c^**	1.63 ± 0.38 ^g^*** (63.03 ± 14.69) ^g^***	1.29 ± 0.29 ^f^* (49.88 ± 11.21) ^f^*
LDL (mmol/L) (mg/dL) ^a^	2.65 ± 0.51 (102.46 ± 19.72)	2.62 ± 0.45 ^c^*** (101.30 ± 17.40) ^c^***	3.80 ± 0.83 ^e^*** (146.99 ± 32.09) ^e^***	2.64 ± 0.60 ^g^*** (102.08 ± 23.20) ^g^***
CRP (mg/L) ^h^	0.60 (0.20–2.10) ^b^*	1.15 (0.25–4.98) ^d^**	1.00 (0.20–2.50) ^g^***	3.95 (1.40–7.83) ^f^***
FRS (skor) ^a^	−3.22 ± 3.56 ^b^***	0.24 ± 5.36 ^c^*^, d^*	2.60 ± 4.13 ^e^***	−2.42 ± 4.24 ^g^***
PA (METs) ^h^	1306.50 (471.00–2826.00)	1479.00 (996.29–2178.00)	1180.50 (683.15–2213.25)	1506.50 (714.25–2692.28)

Data are presented as the mean ± SD and median (IQR). Significance levels are denoted by asterisks * *p* < 0.05, ** *p* < 0.01 and *** *p* < 0.001. a: ANOVA; b: post hoc test between HPT and normal; c: post hoc test between dyslipidemia and HPT; d: post hoc test between obese and HPT; e: post hoc test between dyslipidemia and normal; f: post hoc analysis between obese and normal; g: post hoc test between obese and dyslipidemia; h: Kruskal–Wallis test; N: number; HPT: hypertensive group; SD: standard deviation; IQR: inter-quartile range; BMI: body mass index; bSBP: brachial systolic blood pressure; bDBP: brachial diastolic blood pressure; HR: heart rate; FBS: fasting blood glucose; TC: total cholesterol; TG: triglyceride; HDL: high-density lipoprotein; LDL: low-density lipoprotein; CRP: C-reactive protein; PA: physical activity.

**Table 2:** Central and peripheral vascular function markers for each group.

	Normal (N = 125)	HPT (N = 34)	Dyslipidemia (N = 109)	Obese (N = 52)
PPGF (%) ^a^	52.36 ± 10.73	54.59 ± 11.93	51.08 ± 10.08	52.38 ± 9.07
PWV (m/s) ^a^	6.97 ± 0.82	8.03 ± 1.40 ^b^***^, f^**	7.16 ± 1.10 ^c^**^, g^**	7.37 ± 1.36 ^d^*
AI (%) ^a^	13.86 ± 8.30	21.90 ± 10.57 ^b^***^, h^**	18.20 ± 10.31 ^e^**	16.33 ± 8.56 ^d^*
cSBP (mmHg) ^a^	111.78 ± 10.53	143.77 ± 16.84 ^b^***^, f^***	115.11 ± 10.37 ^c^***^, g^***	115.67 ± 11.15 ^d^***^, i^***
cDBP (mmHg) ^a^	68.99 ± 8.54	86.97 ± 12.80 ^b^***^, f^***	70.71 ± 8.30 ^c^***^, g^***	71.98 ± 8.28 ^d^***^, i^***

Data are presented as the mean ± SD. Significance levels are denoted by asterisks * *p* < 0.05, ** *p* < 0.01 and *** *p* < 0.001. a: ANOVA; b: post hoc test between HPT and normal; c: post hoc test between dyslipidemia and HPT; d: post hoc test between obese and HPT; e: post hoc test between dyslipidemia and normal; f: versus normal after adjustment for age; g: versus HPT after adjustment for age; h: versus normal after adjustment for age, HR, and height; i: versus HPT after adjustment for age; N: number; HPT: hypertensive group; HR: heart rate; PPGF: finger plethysmography fitness index; PWV: pulse wave velocity; AI: augmentation index; cSBP: central systolic blood pressure; aDBP: central diastolic blood pressure.

**Table 3:** Associations between vascular function markers and CVD risk factors among subjects with hypertension, dyslipidemia, and obesity.

	HPT Group	Dyslipidemia Group	Obese Group
PWV	bDBP (*r* = 0.391 *) cSBP(*r* = 0.447 **) cDBP (*r* = 0.391 *) AI (*r* = 0.42 *) BMI (*r* = 0.469 **) FRS (*r* = 0.488 **) CRP (s = 0.425 **) HR (*r* = 0.573 **) Age (*r* = 0.479 **) Height (*r* = −0.463 **)	bSBP (*r* = 0.249 *) bDBP (*r* = 0.378 **) cSBP (*r* = 0.358 **) cDBP (*r* = 0.378 **) AI (*r* = 0.319 **) Age (*r* = 0.255 *) FRS (*r* = 0.316 **) PPGF (*r* = 0.237 *)	bDBP (*r* = 0.308 *) cSBP (*r* = 0.309 *)
AI	bSBP (*r* = 0.352 *) bDBP (*r* = 0.428 *) cSBP (*r* = 0.692 **) cDBP (*r* = 0.428 *) PWV (*r* = 0.420 *) Age (*r* = 0.352 *)	cSBP (*r* = 0.461 **) PWV (*r* = 0.319 **) Age (*r* = 0.510 **) HR (*r* = −0.406 **) PPGF (*r* = −0.349 **) Height (*r* = −0.394 **)	cSBP (*r* = 0.356 *) Age (*r* = 0.522 **) FRS (*r* = 0.372 *) HR (*r* = −0.430 **)
PPGF	bDBP (*r* = −0.383 *) cDBP (*r* = −0.383 *)	Height (*r* = 0.217 *) HDL (*r* = −0.210 *) AI (*r* = −0.349 **) PWV (*r* = −0.237 *) CRP (rho = −0.263 *)	HDL (*r* = −0.360 *) bSBP (*r* = 0.315 *) Height (*r* = 0.339 *)

Data are presented as Pearson’s (*r*) and Spearman’s (rho) correlation coefficients with significance levels denoted by asterisks * *p* < 0.05 and ** *p* < 0.01. HPT: hypertensive group; PWV: pulse wave velocity; AI: augmentation index; bDBP: brachial diastolic blood pressure; cSBP: central systolic blood pressure; cDBP: central diastolic blood pressure; BMI: body mass index; FRS: Framingham risk score; CRP: C-reactive protein; HR: heart rate; bSBP: brachial systolic blood pressure; PPGF: finger plethysmography fitness index; HDL: high-density lipoprotein.

## Data Availability

Data are available upon request due to ethical reason.

## References

[1] Khan T. (2020). Cardiovascular Diseases.

[2] Leon B.M., Maddox T.M. (2015). Diabetes and Cardiovascular Disease: Epidemiology, Biological Mechanisms, Treatment Recommendations and Future Research. World J. Diabetes.

[3] Lorber D. (2014). Importance of Cardiovascular Disease Risk Management in Patients with Type 2 Diabetes Mellitus. Diabetes Metab. Syndr. Obes..

[4] Institute for Public Health (IPH) (2024). Technical Report National Health and Morbidity Survey (NHMS) 2023: Non-Communicable Diseases and Healthcare Demand, Malaysia.

[5] Thangiah N., Su T.T., Chinna K., Jalaludin M.Y., Mohamed M.N.A., Majid H.A. (2021). Longitudinal assessment between lifestyle-related risk factors and a composite cardiovascular disease (CVD) risk index among adolescents in Malaysia. Sci. Rep..

[6] Chen A., Azuan N.B.R., Ooi Y.B.H., Khor B.H. (2024). Nutritional status and dietary fatty acid intake among children from low-income households in Sabah: A cross-sectional study. Hum. Nutr. Metab..

[7] Regnault V., Lacolley P., Laurent S. (2024). Arterial stiffness: From basic primers to integrative physiology. Annu. Rev. Physiol..

[8] Higashi Y. (2024). Noninvasive Assessment of Vascular Function: From Physiological Tests to Biomarkers. JACC Asia..

[9] Van Bortel L.M., Laurent S., Boutouyrie P., Chowienczyk P., Cruickshank J.K., De Backer T., Filipovsky J., Huybrechts S., Mattace-Raso F.U., Protogerou A.D. (2012). Expert consensus document on the measurement of aortic stiffness in daily practice using carotid-femoral pulse wave velocity. J. Hypertens..

[10] Aminuddin A., Salamt N., Ahmad Fuad A.F., Chin K.Y., Ugusman A., Soelaiman I.N., Wan Ngah W.Z. (2019). Vascular dysfunction among Malaysian men with increased BMI: An indication of Synergistic effect of free testosterone and inflammation. Medicina.

[11] Marshall A.G., Neikirk K., Afolabi J., Mwesigwa N., Shao B., Kirabo A., Reddy A.K., Hinton Jr A. (2024). Update on the use of pulse wave velocity to measure age-related vascular changes. Curr. Hypertens. Rep..

[12] Aminuddin A., Zakaria Z., Chellapan K., Ugusman A., Norizam S., Nordin N.A.M. (2016). The Assessment of Finger Photoplethysmography Fitness Index (PPGF) among Young Men with Cardiovascular Disease Risk Factors: A Cross Sectional Study. Med. Health.

[13] Chrousos G.P., Papadopoulou-Marketou N., Bacopoulou F., Lucafò M., Gallotta A., Boschiero D. (2022). Photoplethysmography (PPG)-determined heart rate variability (HRV) and extracellular water (ECW) in the evaluation of chronic stress and inflammation. Hormones.

[14] Vlachopoulos C., Xaplanteris P., Aboyans V., Brodmann M., Cífková R., Cosentino F., De Carlo M., Gallino A., Landmesser U., Laurent S. (2015). The Role of Vascular Biomarkers for Primary and Secondary Prevention. A Position Paper from the European Society of Cardiology Working Group on Peripheral Circulation. Endorsed by the Association for Research into Arterial Structure and Physiology (ARTERY) Society. Atherosclerosis.

[15] Tomiyama H., Matsumoto C., Shiina K., Yamashina A. (2016). Brachial-Ankle PWV: Current Status and Future Directions as a Useful Marker in the Management of Cardiovascular Disease and/or Cardiovascular Risk Factors. J. Atheroscler. Thromb..

[16] Feola M., Testa M., Ferreri C., Rosso G., Rossi A., Ruocco G. (2019). The Analysis of Arterial Stiffness in Heart Failure Patients in Comparison with Healthy Subjects and Patients with Cardiovascular Risk Factors. J. Clin. Med..

[17] Janner J.H., Godtfredsen N.S., Ladelund S., Vestbo J., Prescott E. (2012). The Association between Aortic Augmentation Index and Cardiovascular Risk Factors in a Large Unselected Population. J. Hum. Hypertens..

[18] Challoner A.V.J. (1979). Photoelectric plethysmography for estimating cutaneous blood flow. J. Bone Jt. Surg. Am..

[19] Chellappan K., Mohd Ali M.A., Zahedi E., Abu Osman N.A., Ibrahim F., Wan Abas W.A.B., Abdul Rahman H.S., Ting H.N. (2008). An Age Index for Vascular System Based on Photoplethysmogram Pulse Contour Analysis. IFMBE Proceedings, Proceedings of the 4th Kuala Lumpur International Conference on Biomedical Engineering 2008, Kuala Lumpur, Malaysia, 25–28 June 2008.

[20] Aminuddin A., Tan I., Butlin M., Avolio A.P., Kiat H., Barin E., Nordin N.A.M.M., Chellappan K. (2018). Effect of Increasing Heart Rate on Finger Photoplethysmography Fitness Index (PPGF) in Subjects with Implanted Cardiac Pacemakers. PLoS ONE.

[21] Zainudin S., Daud Z., Mohamad M., Tan A.T.B., Wan Mohamed W.M.I. (2011). A Summary of the Malaysian Clinical Practice Guidelines on Management of Obesity 2004. J. ASEAN Fed..

[22] Craig C.L., Marshall A.L., Sjöström M., Bauman A.E., Booth M.L., Ainsworth B.E., Pratt M. (2003). International physical activity questionnaire: 12-Country reliability and validity. Med. Sci. Sports Exerc..

[23] Ring M., Eriksson M.J., Zierath J.R., Caidahl K. (2014). Arterial Stiffness Estimation in Healthy Subjects: A Validation of Oscillometric (Arteriograph) and Tonometric (SphygmoCor) Techniques. Hypertens. Res..

[24] Diaz A., Zócalo Y., Bia D., Cabrera Fischer E. (2018). Reference Intervals of Central Aortic Blood Pressure and Augmentation Index Assessed with an Oscillometric Device in Healthy Children, Adolescents, and Young Adults from Argentina. Int. J. Hypertens..

[25] Gkaliagkousi E., Gavriilaki E., Triantafyllou A., Nikolaidou B., Anyfanti P., Koletsos N., Vamvakis A., Dipla K., Lazaridis A., Douma S. (2018). Asymmetric Dimethylarginine Levels Are Associated with Augmentation Index across Naïve Untreated Patients with Different Hypertension Phenotypes. J. Clin. Hypertens..

[26] Martinez-Quinones P., McCarthy C.G., Watts S.W., Klee N.S., Komic A., Calmasini F.B., Priviero F., Warner A., Chenghao Y., Wenceslau C.F. (2018). Hypertension Induced Morphological and Physiological Changes in Cells of the Arterial Wall. Am. J. Hypertens..

[27] Guzik T.J., Touyz R.M. (2017). Oxidative Stress, Inflammation, and Vascular Aging in Hypertension. Hypertension.

[28] Harvey A., Montezano A.C., Lopes R.A., Rios F., Touyz R.M. (2016). Vascular Fibrosis in Aging and Hypertension: Molecular Mechanisms and Clinical Implications. Can. J. Cardiol..

[29] Mitchell G.F. (2014). Arterial Stiffness and Hypertension: Chicken or Egg?. Hypertension.

[30] Wagenseil J.E., Mecham R.P. (2012). Elastin in large artery stiffness and hypertension. J. Cardiovasc. Transl. Res..

[31] Weber M.A. (2009). Treatment of Patients with Hypertension and Arthritis Pain: New Concepts. Am. J. Med..

[32] Laugesen E., Olesen K.K., Peters C.D., Buus N.H., Maeng M., Botker H.E., Poulsen P.L. (2022). Estimated pulse wave velocity is associated with all-cause mortality during 8.5 years follow-up in patients undergoing elective coronary angiography. J. Am. Heart Assoc..

[33] Lee G.K., Lee L.C., Liu C.W., Lim S.L., Shi L.M., Ong H.Y., Lim Y.T., Yeo T.C. (2010). Framingham risk score inadequately predicts cardiac risk in young patients presenting with a first myocardial infarction. Ann. Acad. Med. Singap..

[34] Schettini I.V.G., Rios D.R.A., Figueiredo R.C. (2023). Effect of different classes of antihypertensive drugs on arterial stiffness. Curr. Hypertens. Rep..

[35] Baharudin A., Ambak R., Othman F., Michael V., Cheong S.M., Mohd Zaki N.A., Abdul Aziz N.S., Mohd Sallehuddin S., Ganapathy S.S., Palaniveloo L. (2021). Knowledge, attitude and behaviour on salt intake and its association with hypertension in the Malaysian population: Findings from MyCoSS (Malaysian Community Salt Survey). J. Health Popul. Nutr..

[36] Vlachopoulos C., Aznaouridis K., Stefanadis C. (2010). Prediction of cardiovascular events and all-cause mortality with arterial stiffness: A systematic review and meta-analysis. J. Am. Coll. Cardiol..

[37] Desamericq G., Tissot C.M., Akakpo S., Tropeano A.I., Millasseau S., MacQuin-Mavier I. (2015). Carotid-Femoral Pulse Wave Velocity Is Not Increased in Obesity. Am. J. Hypertens..

[38] Lurbe E., Torro I., Garcia-Vicent C., Alvarez J., Fernández-Fornoso J.A., Redon J. (2012). Blood Pressure and Obesity Exert Independent Influences on Pulse Wave Velocity in Youth. Hypertension.

[39] Kulsum-Mecci N., Goss C., Kozel B.A., Garbutt J.M., Schechtman K.B., Dharnidharka V.R. (2016). Effects of Obesity and Hypertension on Pulse Wave Velocity in Children. J. Clin. Hypertens..

[40] Son W.-M., Kim D.-Y., Kim Y.-S., Ha M.-S. (2017). Effect of Obesity on Blood Pressure and Arterial Stiffness in Middle-Aged Korean Women. Osong Public Health Res. Perspect..

[41] Corden B., Keenan N.G., de Marvao A.S., Dawes T.J., DeCesare A., Diamond T., Durighel G., Hughes A.D., Cook S.A., O’Regan D.P. (2013). Body fat is Associated with Reduced Aortic Stiffness Until Middle Age. Hypertension.

[42] Alpert M.A., Omran J., Bostick B.P. (2016). Effects of Obesity on Cardiovascular Hemodynamics, Cardiac Morphology, and Ventricular Function. Curr. Obes. Rep..

[43] Munir S., Jiang B., Guilcher A., Brett S., Redwood S., Marber M., Chowienczyk P. (2008). Exercise Reduces Arterial Pressure Augmentation through Vasodilation of Muscular Arteries in Humans. Am. J. Physiol.-Heart Circ. Physiol..

[44] Daniels S.R. (2012). Obesity, vascular changes, and elevated blood pressure. J. Am. Coll. Cardiol..

[45] Kim Y.-K. (2006). Impact of the Metabolic Syndrome and Its Components on Pulse Wave Velocity. Korean J. Intern. Med..

[46] Lee C.-J., Wang J.-H., Chen Y.-C., Chen M.-L., Yang C.-F., Hsu B.-G. (2014). Serum Osteopontin Level Correlates with Carotid-Femoral Pulse Wave Velocity in Geriatric Persons. BioMed Res. Int..

[47] Nägele M.P., Barthelmes J., Ludovici V., Cantatore S., Frank M., Ruschitzka F., Flammer A.J., Sudano I. (2018). Retinal Microvascular Dysfunction in Hypercholesterolemia. J. Clin. Lipidol..

[48] Tsai W.-C., Chen J.-Y., Wang M.-C., Wu H.-T., Chi C.-K., Chen Y.-K., Chen J.-H., Lin L.-J. (2005). Association of Risk Factors with Increased Pulse Wave Velocity Detected by a Novel Method Using Dual-Channel Photoplethysmography. Am. J. Hypertens..

[49] Wilkinson I.B., Prasad K., Hall I.R., Thomas A., Maccallum H., Webb D.J., Frenneaux M.P. (2002). Increased central pulse pressure and augmentation index in subjects with hypercholesterolemia. J. Am. Coll. Cardiol..

[50] Xiang H., Xue Y., Wang J., Weng Y., Rong F., Peng Y., Ji K. (2020). Cardiovascular Alterations and Management of Patients with White Coat Hypertension: A Meta-Analysis. Front. Pharmacol..

[51] Dogdus M., Akhan O., Ozyasar M., Yilmaz A., Altintas M.S. (2018). Evaluation of Arterial Stiffness Using Pulse Wave Velocity and Augmentation Index in Patients with Chronic Venous Insufficiency. Int. J. Vasc. Med..

[52] Moon S.-H., Moon J.-C., Heo D.-H., Lim Y.-H., Choi J.-H., Kim S.-Y., Kim K.-S., Joo S.-J. (2015). Increased Pulse Wave Velocity and Augmentation Index after Isometric Handgrip Exercise in Patients with Coronary Artery Disease. Clin. Hypertens..

[53] Laurent S., Agabiti-Rosei C., Bruno R.M., Rizzoni D. (2022). 2022. Microcirculation and Macrocirculation in Hypertension: A Dangerous Cross-Link?. Hypertension.

[54] Chung J.W., Lee Y.S., Kim J.H., Seong M.J., Kim S.Y., Lee J.B., Ryu J.K., Choi J.Y., Kim K.S., Chang S.G. (2010). Reference Values for the Augmentation Index and Pulse Pressure in Apparently Healthy Korean Subjects. Korean Circ. J..

[55] Otsuka T., Kawada T., Ibuki C., Kusama Y. (2009). Obesity as an Independent Influential Factor for Reduced Radial Arterial Wave Reflection in a Middle-Aged Japanese Male Population. Hypertens. Res..

[56] Aminuddin A., Chellappan K., Maskon O., Zakaria Z., Karim A.A., Ngah W.Z., Nordin N.A.M. (2014). Augmentation Index is a Better Marker for Cardiovascular Risk in Young Malaysian Males: A Comparison of Involvement of Pulse Wave Velocity, Augmentation Index, and C-Reactive Protein. Saudi Med. J..

[57] Laurent S., Cockcroft J., Van Bortel L., Boutouyrie P., Giannattasio C., Hayoz D., Pannier B., Vlachopoulos C., Wilkinson I., Struijker-Boudier H. (2007). European Network for Non Invasive Investigation of Large Arteries. Abridged version of the expert consensus document on arterial stiffness. Artery Res..

[58] Chellappan K. (2009). Noninvasive Vascular Risk Prediction by Photoplethysmogram Analysis. Ph.D. Thesis.

[59] Sari I., Soydinc S., Davutoglu V., Sezen Y., Aksoy M. (2008). Uncomplicated Diabetes Mellitus Is Equivalent for Coronary Artery Disease: New Support from Novel Angiographic Myocardial Perfusion-Myocardial Blush. Int. J. Cardiol..

[60] Cecelja M., Chowienczyk P. (2009). Dissociation of Aortic Pulse Wave Velocity with Risk Factors for Cardiovascular Disease Other than Hypertension: A Systematic Review. Hypertension.

[61] Muhajir M., Aminuddin A., Ugusman A., Salamt N., Asmawi Z., Zulkefli A.F., Azmi M.F., Chellappan K., Nordin N.A.M.M. (2018). Evaluation of Finger Photoplethysmography Fitness Index on Young Women with Cardiovascular Disease Risk Factors. Sains Malays.

[62] Heidemann B.E., Koopal C., Bots M.L., Asselbergs F.W., Westerink J., Visseren F.L. (2021). The relation between VLDL-cholesterol and risk of cardiovascular events in patients with manifest cardiovascular disease. Int. J. Cardiol..

